# Return-to-work-experts for inpatient treatment of patients with mental illnesses– a proof-of-concept-study (RETURN): the study protocol

**DOI:** 10.1186/s12888-020-02504-4

**Published:** 2020-04-19

**Authors:** Lina Riedl, Daniela Blank, Monika Kohl, Anne Lang, Victoria Kehl, Peter Brieger, Johannes Hamann

**Affiliations:** 1 Department of Psychiatry and Psychotherapy of Technische Universität München, School of Medicine, Ismaninger Str.22, 81675 Munich, Germany; 2 kbo-Isar-Amper-Klinikum, Academic Teaching Hospital LMU Munich, Munich-Haar, Germany; 3 Academic Teaching Hospital LMU, Munich, Germany; 4 Department of Psychiatry and Psychotherapie, Technische Universität München, School of Medicine, Munich, Germany

**Keywords:** Return to work, Mental health, Hospitals, Psychiatric

## Abstract

**Background:**

Patients with mental illnesses often have massive difficulties returning to work after inpatient treatment at a psychiatric clinic and are often at risk of losing their jobs. The psychosocial support for this patient group at the interface of clinic/outpatient care is often insufficient.

**Methods/design:**

The RETURN-study prospectively assesses and surveys 200 patients with mental disorders in a cluster randomized intervention study, i.e. treatment teams and patients from intervention wards receive a return-to-work (RTW) intervention. Patients in control wards obtain treatment as usual (TAU). Pairs of comparable wards (similar patient population, similar staff density) have been identified and then randomized for control and intervention (*n* = 14 for each condition). On intervention wards return-to-work experts (RTW experts) who focus treatment on the workplace-related needs of patients with mental illnesses have been established. These RTW experts ensure the use of available resources within the framework of work-related discharge management and should lead to a more successful return to the workplace.

The days at work in the year after release will be evaluated in a mixed methods approach as well as the return rate in the year after release, disability days in the year after return, relapse rate after 12 months, cost-benefit ratio of the intervention, analysis of the predictors / barriers for a successful return to the workplace (e.g. psychopathology, cognition, stigma, social-psychiatric support, company support, etc.), possibilities to implement the concept of RTW experts in standard psychiatric care (TAU - treatment as usual), the impact of the RTW experts’ approach on the treatment process in standard psychiatric care.

**Discussion:**

This approach is already internationally established in the field of somatic rehabilitation and supported employment [Am J Psychiatry 171:1183–90, 2014; Lancet 370:1146–52, 2007; Cochrane Database Syst Rev, doi:10.1002/14651858.CD006237.pub3, 2014]; the innovative aspect of this project is to implement and evaluate it in standard psychiatric care in Germany. This project requires no new interventions to be developed and tested, as the techniques of the case manager/job coach is applied to the field of return to work.

**Trial registration:**

The study was registered in Deutsches Register Klinische Studien searchable via its Meta-registry (http://apps.who.int/trialsearch/), Trial registration number: DRKS00016037, Date of registration: 21/12/2018, URL of trial registry record.

## Background

Mental illnesses are among the most common diseases worldwide. In Germany, 28% of the population aged 18–79 have suffered from a mental illness within the last 12 months [1]. Accordingly, cases of inability to work caused by mental illness are high. In particular the days of inability to work have been increasing in recent years. Mental illness is now the third most frequent diagnostic group for sick leave or inability to work [2, 3]. The consequences for health insurers, companies and national economy are expenditures in billions of Euro, not including the additional indirect costs, especially for early retirement. Yet, mental illness is the most common cause of early retirement in Germany [4].

Mental illness and the ability to work interact in many different ways. Therefore, many patients with severe mental illness (e.g. schizophrenia or bipolar affective disorder) do not manage to find and keep regular employment. In addition, the number of cases of mental illness (usually depressive disorders) in Germany have risen for years as have the days absent from work [5, 6].

On the other hand, work and employment may have positive effects on the course of illness [7–9]. There is an undisputable salutogenetic aspect of work [10]. Work in general has a beneficial effect on health. Randomized studies demonstrated that mentally ill people who were supported as early as possible in their job search and in returning to work succeed more often than patients undergoing a long rehabilitation phase first [11]. In addition, these studies showed that permanent employment (competitive employment) increases quality of life and reduces hospital days [12].

Accordingly, obtaining and maintaining jobs for people with mental illness is challenging on the one hand and an important prerequisite for a good prognosis on the other. A loss of employment due to mental illness is not only associated with negative consequences for those affected (i.e. missing day structure), but is a serious societal problem as well, due to resulting follow-up costs (sickness benefits, unemployment benefits, reduced social contributions etc.)

### Acute psychiatric crises, inpatient treatment and return to work

A particularly vulnerable time is that of an acute mental health crisis, which requires inpatient hospital treatment. It tears those affected out of their employment relationships, sometimes over longer periods of time. A successful reintegration of those patients after the acute episode of illness would therefore be an important prognostic factor for the further course of the disease. A failed reintegration in contrast would be a high risk factor for longer incapacity to work, long payment of sickness benefit, unemployment and a less favourable course of the disease.

Present data shows that it is a frequent health (or social) problem. In 2010 about 800,000 psychiatric inpatient cases were documented in Germany [13]. About 20% (= 160,000 people) of these cases were working patients [14]. Up to 30% of these patients did not return to work after discharge from hospital [14] and of those returning it was unclear how long they were able to successfully resume their employment relationships. Considering the related social decline and associated reduction in participation in social life, these numbers are alarming [15, 16].

### Reasons for unsatisfactory return to work after psychiatric inpatient treatment

Reasons for the frequent job losses after inpatient psychiatric treatment are multifactorial and often mutually dependent. In particular, awareness for aspects of work is often too low in the context of psychiatric in- or out-patient treatment. Furthermore, the lack of support of those affected when returning to work, the dealing with the disease, especially in the context of work, as well as how employers deal with the affected people or the working conditions may play a role. In the context of psychiatric inpatient treatment, the salutogenetic effects of work are given too little consideration. The fact that returning to work can not only be a treatment goal, but also a therapeutic action, is often neglected in the reality of psychiatric care. Currently, a corresponding formalized procedure is insufficiently integrated into the processes of psychiatric hospitals.

Psychosocial counselling and support are also part of the treatment of the disease, especially with regard to the return to work process. Although the current German S3 guideline “Psychosocial therapies for severe mental disorders” emphasizes the importance of the return to work process for the patients, many clinics often fail to appropriately prepare patients for routine reasons [17]. Established support such as re-entry counselling and structured reintegration measures (e.g. BEM, gradual reintegration) are currently only implemented for a fraction of patients and, above all, are not supported across the various interfaces [14]. As a result, the return to work remains fearful for many patients [14, 18] and the actions that may be initiated in the clinic may not be continued after discharge from inpatient care.

### Aim and research questions

The aim of the planned project is to establish a psychosocial case-management intervention (return-to-work-experts) that supports patients at the interface between inpatient treatment and return to work and to estimate its effects in a cluster-randomized study.

**Fig. 1:**
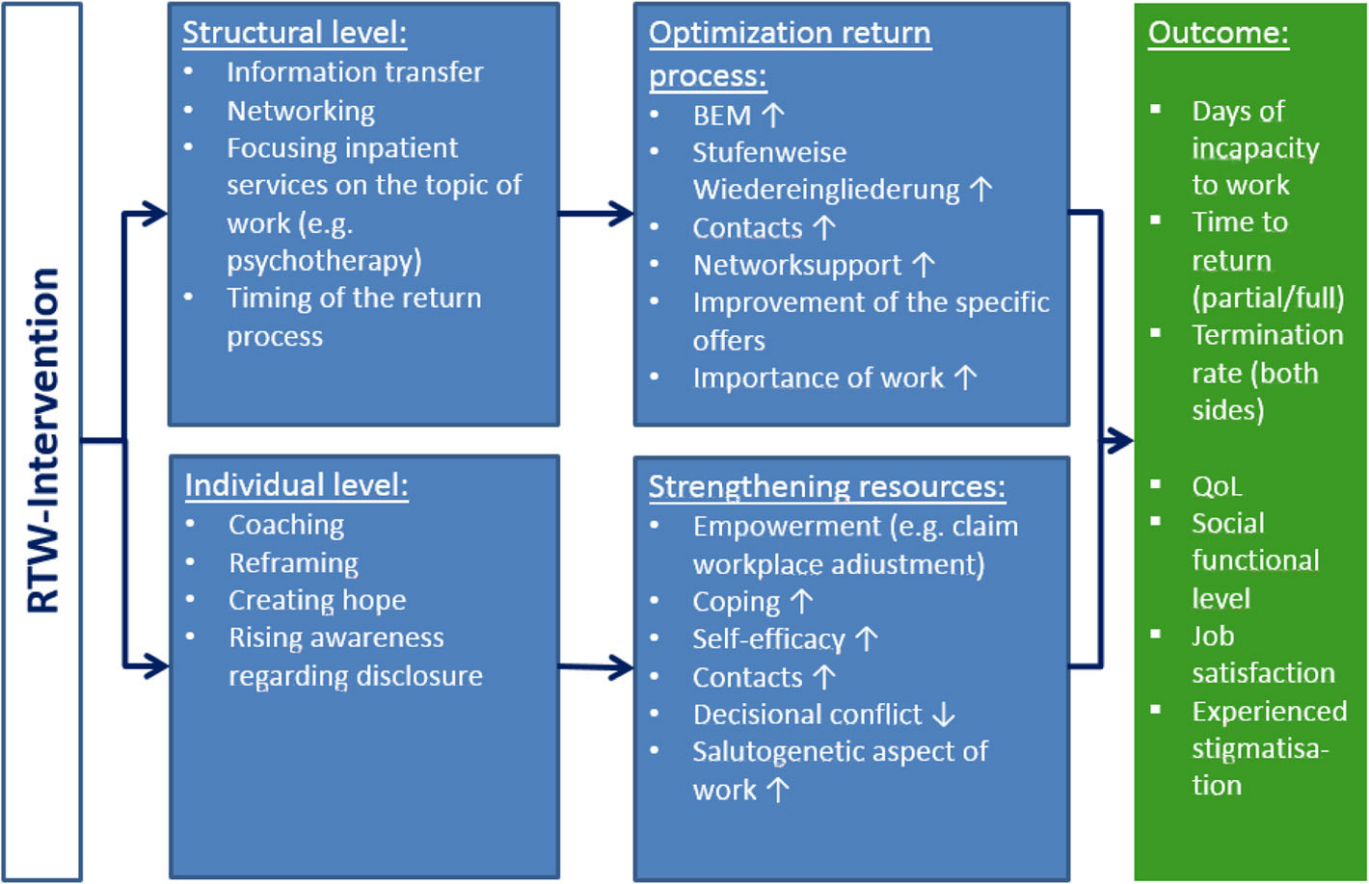
German terms and their translation: BEM = Betriebliches Eingliederungsmanagement (Corporate integration management), Stufenweise Wiedereingliederung (gradual reintegration)

We presume that the intervention (return-to-work-experts, details see methods) will optimize care of the affected patients with regard to improving their return to work, thereby focusing on the interface between in-patient treatment and outpatient care (“occupational discharge management”). The chain of action on the structural and the individual level is visualized in Fig. 1.

We expect improvements in the number of patients who will be able to return to work and also in the number of days at work in the year after release from hospital. This could also result in substantial economic savings (for example, sickness benefit, loss of productivity, etc.). Mediators of these expected improvements are increased numbers of patients for whom specific supportive measures (e.g., work therapy, gradual reintegration, BEM processes, etc.) are initiated. We assume that the proposed intervention primarily will increase the implementation of measures already existing in routine care and will help to overcome the interface problem at the time of inpatient discharge.

## Methods

### Study design

The study is designed as a multi-center, cluster-randomized controlled trial in acute psychiatric wards addressing inpatients suffering from a psychiatric disorder. RTW-experts will be introduced on wards chosen for the intervention, while on control wards treatment as usual will be continued.

### Setting and participants

The study will be implemented on *n* = 28 acute wards (=clusters) in seven psychiatric hospitals in the greater Munich area. Eligibility criteria for the wards to be included are: acute psychiatric ward in one of the participating hospitals, patients with psychiatric disorders are commonly treated on these wards.

All patients treated on these wards and on admission to the ward, each patient fulfilling the inclusion criteria will be consecutively offered participation in the trial.

#### Inclusion criteria

· Age 18–60 years.

· Diagnosis of a mental illness [ICD-10 Chapter F2–4, F6 (we assume that the majority of patients with existing employment relationships suffer from an affective disease [14]).

· In a contractual employment relationship, in which they can be reintegrated.

· Psychopathologically capable of reflecting on work in general and on their own work.

#### Exclusion criteria

· Cognitive impairment.

· Insufficient proficiency in German language.

· Main diagnosis of an organic mental disorder (F0), substance abuse (F1) or an eating disorder (F5).

#### Randomization and blinding

We will determine pairs of comparable wards (number of patients, distribution of diagnoses, staff etc.) and then randomize one ward of each pair to the intervention and one ward to the control condition. While the principal investigators will determine the paired wards, the statistical institute of our department (IMedIS) will do the randomization.

Randomization takes place at cluster level (*n* = 14 wards each to the intervention group or control group) to minimize contamination effects [19].

As to the nature of the intervention (implementation of RTW-experts) there will be no blinding.

To avoid selection bias, all patients fulfilling inclusion criteria will be recruited consecutively in the intervention and control group. This process is monitored by the study center and will be reported in form of a CONSORT diagram. There will be special emphasis on documenting how many patients (and for what reasons) did not consent to participate in the trial.

### Intervention and control condition

The intervention consists of the implementation of Return to Work experts in the participating acute care units. The RTW experts (social workers, who in the framework of the RETURN study are added to existing resources) support patients and mental health care professionals on the wards in all areas related to the possible return of patients to their workplace. The RTW experts will thereby act as case managers, who usually first clarify the specific needs of the clients (i.e. participating patients who might return to an existing workplace), subsequently communicate these needs to all people involved in the treatment process and initiate and accompany a corresponding treatment path. The services of the RTW expert can be claimed by the patients for up to half a year. In order to guarantee a minimum of intervention, it is planned that the RTW experts meet at least twice – one appointment during the inpatient stay and one after release. A maximum of intervention is not defined. Above all, existing support services (e.g. work therapy, cognitive training, socio-educational counseling, etc.) should be activated for the individual patient. However, if there is a limitation of resources, more specific needs or issues (for example, discussions with the patient’s employer, Integration Services, etc.), the RTW expert can also take personal action and support the patients accordingly. In addition, the RTW expert is to be active at the interface between discharge and reintegration and to assist the patient even after discharge in the activation of occupational and outpatient psychiatric-psychotherapeutic support measures. The RTW experts thus assume the function of a job-related discharge management.

The approach of the RTW experts is guideline-based. The RTW experts will be specifically trained. For this purpose, a guideline and a training program for the RTW experts has been established together with the Advisory Board in the first project phase (Kohl et al. in preparation), and will be evaluated within the framework of the study and then revised if necessary, so that the guideline and RTW expert training can be published at the end of the project.

Staff (and patients) of the control wards will act under “treatment as usual” (TAU) conditions. To avoid contamination bias as far as possible (i.e. staff or patients from control wards getting to know about RETURN), wards were chosen in a way so that there is no overlap in personnel and that there is no regular patient exchange between wards.

### Data collection

The same data at the same time points will be collected in the intervention and control group (Table 1). Data collection will take place at five times during the inpatient stay and after discharge.

**Table 1:** Planned data collection

	T0 (shortly after admission to hospital)	T1 (shortly before release from hospital)	T2 (3 months after release)	T3 (6months after release)	T4 (12 months after release)
Patient					
Demographics including information on					
School and vocational training	x				
Employment relationsship	x				
Business, enterprise (industry)	x				
Business, enterprise (size)	x				
Motivation	x	x	x	x	x
Anxiety, confidence	x	x			
Days of incapacity to work	x		x	x	x
COPSOQ	x			x	
COPSOQ (additional information)	x				
Self-stigma of mental illness scale short form – self-concurrence	x	x		x	
Cognitive appraisal of stigma-related stress	x	x		x	
Questions concerning disclosure of the disease at the workplace including					
Questions on the decision-making phase	x	x		x	
Questions on the decision to disclose	x	x		x	
Questions on the attitude towards disclosure	x	x		x	
Decisional Conflict scale	x	x		x	
Cognitive performance level		x			
CSSRI	x	x	x	x	x
Preparation of the return through the hospital		x			
Preparation of the return through the employer		x			
Preparation of the return through the RTW expert (intervention group only)		x		x	
Questions on the existing help network		x		x	x
Content of the employer support				x	
Evaluation of the employer support				x	
Evaluation of the RTW expert support (intervention group only)				x	
EuroQOL - Gesundheitsbarometer	x	x		x	
EQ-5D		x		x	x
Self-efficacy expectation (RTW-SE)		x			
Resilience (RS-13)					x
Patient Health Questionnaire (PHQ-D)		x			
Days at work			x	x	x
Days of incapacity for work			x	x	x
Days of partial return to work			x	x	x
Questions on discrimination at the workplace				x	
Coping				x	
Attending physician					
CGI	x	x			x
GAF	x	x			x
ICD Diagnosis and medical history	x	x			x
HoNOS		x			
Prediction on return to work		x			
RTW expert (intervention group only)					
Type and scope of workplace-related support		x		x	
Evaluation of workplace-related support		x		x	
Prediction on return to work		x			
Quality of relationship		x		x	
Return to work				x	
Questions concerning the use of communication media				x	
Open summary				x	

### Study status and study sites

At the moment of submission participant recruitment is ongoing at all seven study sites: Munich district hospital (kbo-Isar-Amper-Klinikum) with three locations (Klinik Nord, Klinik Fürstenfeldbruck, Klinik Ost), two Munich university hospitals, Klinikum der Universität München, Department of Psychiatry and Psychotherapie, Department of Psychiatry and Psychotherapy of Technische Universität München, Max-Planck-Institute for Psychiatry in Munich and one district hospital (kbo-Lech-Mangfall-Klinik) in the greater Munich area.

#### Primary outcome

The scientific rationale is that the establishment of return-to-work (RTW) experts for inpatient mental health patients focuses on the workplace needs of these patients, leading to a better usage of existing resources as part of a work-related discharge management, and thus to a more successful return to the workplace.

The primary objective is to display this improved return to the workplace after in-patient psychiatric care as a group difference (intervention vs. control) using days in work and days of incapacity to work during 1 year after discharge from hospital.

#### Secondary outcomes

As outlined above an improved return to work following an in-patient psychiatric care treatment of patients may also result in a shorter period of **time to return to work** (partial/full), lower **termination rates** (both sides), a higher **quality of life** (EQ-5D, [20]), a higher **social functional level** (Mini-ICF-APP, [21]; Global Assessment of Functioning (GAF), [22]), a higher **job satisfaction** (German version of the Copenhagen psychosocial questionnaire (COPSOQ), [23]), a different **perception of stigmatization** (Self-stigma of mental illness scale – short form – subscale self-concurrence, [24]; Cognitive appraisal of stigma-related stress, [25]), lower **decisional conflict in regard to disclosure of the mental illness** (Decisional conflict Scale, [26]) and probable reduced **relapse rates**.

The basis for estimating the **cost of the illness** and the **health economic evaluation** of the RTW intervention are the medical and psychosocial health services made use of by the subjects and the number of days of incapacity for work for the whole study period.

The days of incapacity to work of the patients are on the one hand directly requested by the patients and their practitioners. It is envisaged that the patient’s details will be checked against those of some health insurance companies in order to assess the extent to which the data match.

#### Qualitative data

To generate a better understanding of the return-to-work process and potential mechanisms of the intervention qualitative data will also be obtained. The evaluation uses multiple instruments and a mixed-methods design: Face-to-face, audio-taped, semi-structured interviews (individual and focus groups) and analysis of medical records and RTW-expert documentation. The survey involves patients (approx. *n* = 20) and as far as possible all staff members who are concerned with the daily practices of return to work (e.g. return-to-work-experts, social worker, physicians, psychologists). Data collection, data analysis and theoretical development will be based on the fundamental process of the grounded theory. Among other things, this includes theoretical sampling, the coding of the data and the application of the coding paradigm [27, 28].

### Treatment adherence

To assess treatment adherence, we will document whether all patients got an RTW intervention, the contact frequency, to what extent single units of the intervention were implemented for a patient (i.e. coaching sessions) and what the RTW experts did. In addition, we will use a qualitative approach in the intervention group to assess various aspects of the process of RETURN as well as barriers and facilitators of RETURN.

### Sample size calculation and statistical analysis

The aim is to obtain a sample of *n* = 28 clusters (i.e. psychiatric acute wards) with a total of 200 patients (8–10 per cluster). For this, about 2000 patients need to be screened (Mernyi et al., 2018).

In order to be able to demonstrate a significant result with a power of 80% assuming a small to medium effect size (0.5), a total sample size of 128 patients is required (two-sided independent samples t-test, α = 0.05). With regard to expected drop-outs (drop-out rate 30–40%), this minimum number of cases is planned to be exceeded by 72 patients. Thus, a total of 200 patients are to be included (100 in each group).

The primary analysis will be a comparison of days in work at T4 between the intervention and control groups. To assess the effect of the intervention on the continuous primary outcome, a random effects linear regression model will be fitted with ward (cluster) as a random effect term and intervention group as a fixed effect. The point estimate for the intervention effect will be reported together with the corresponding 95% confidence interval. A *p*-value < 0.05 will be considered as providing statistical significant evidence of a group difference. A per protocol approach will be taken to the analysis, i.e. patients in intervention clusters will be analyzed in this group, if they had contact with the RTW-expert at least once during their inpatient stay and once after discharge.

Exploratory analyses will be performed to assess the effect of the intervention on the secondary outcome measures. Random effect linear models will be fitted to the continuous secondary outcome measures, analogous to the primary analysis. For binary secondary outcome measures, logistic regression models using GEEs will be fitted.

The health economic analysis is based on the net benefit approach. To analyze the cost-effectiveness of the RWT intervention, the incremental cost-effectiveness ratio (ICER) is determined for gaining a QALY compared to routine treatment. The estimation of ICER stochastic uncertainty is done by nonparametric bootstrapping and the determination of the cost-effectiveness acceptance curve [29].

### Ethics, informed consent procedure and trial registration

The trial has been approved by the local review board (Ethikkommission der Technischen Universität München). The ethics approval covers all study sites under the German Law (Gesundheitsdienst- und Verbraucherschutzgesetz (GDVG) vom 24. Juli 2003; GVBl. S. 452, 752, BayRS 2120–1-U/G; last changed through § 1 Abs. One hundred fourty-five der Verordnung vom 26. März 2019 (GVBl. S. 98)).

All patients will be informed about the general purpose of the trial (i.e. that return to work patterns will be compared between different wards) but not about randomization and about the intervention condition and then asked for their informed consent. The trial has been registered at Deutsches Register Klinischer Studien (DRKS00016037), searchable via its Meta-registry (http://apps.who.int/trialsearch/).

## Discussion

There has been an increasing awareness of the interplay between work and mental health in recent years. However, the group of patients suffering from (severe) mental illnesses that require hospitalization and their potential return to their workplaces has been neglected. The RETURN study aims to establish and evaluate a pragmatic intervention that may improve the return to work process of these patients.

This approach is already internationally established in the field of somatic rehabilitation and supported employment [30–32]; the innovative aspect of this project is to implement and evaluate it in standard psychiatric care in Germany. This project requires no new interventions to be developed and tested, as the techniques of the case manager/job coach is applied to the field of return to work.

The inclusion of patients with severe mental illnesses (including those with schizophrenia, bipolar disorder, borderline personality etc.), the controlled design with cluster randomization and the duration of the follow-up period are methodical strengths. In addition, we intend to use a broad recruitment basis (the great majority of psychiatric hospitals in the greater Munich area) to enhance the generalizability of the results. Finally, the mixed-methods approach will allow deep insight into potential barriers and facilitators of the implementation of our intervention.

The heterogeneity of the participants with regard to diagnoses, illness severity and working environment as well as the settings (open vs. closed wards, specialized vs. non-specialized wards) could limit the significance of the results. In addition, the special feature of the study – an intervention in the form of a case management that focuses on work - can only be standardized to a certain extent.

To conclude we expect the RTW-experts to better focus inpatient treatment on the specific needs of patients who are still competitively employed and thereby improve return to work patterns.

## Data Availability

Not applicable.

## Abbreviations

BEM: Betriebliches eingliederungs management (Corporate integration management)
CGI: Clinical global impression
COPSOQ: Copenhagen psychosocial questionnaire
CSSRI: Client sociodemographic and service receipt inventory
DRKS: Deutsches register klinischer studien
GAF: Global assessment of functioning
HoNOS: Health of the nation outcome scales
ICD: International statistical classification of diseases and related health problems
RTW: Return-to-work
